# MUC16 provides immune protection by inhibiting synapse formation between NK and ovarian tumor cells

**DOI:** 10.1186/1476-4598-9-11

**Published:** 2010-01-20

**Authors:** Jennifer AA Gubbels, Mildred Felder, Sachi Horibata, Jennifer A Belisle, Arvinder Kapur, Helen Holden, Sarah Petrie, Martine Migneault, Claudine Rancourt, Joseph P Connor, Manish S Patankar

**Affiliations:** 1 Department of Obstetrics and Gynecology, University of Wisconsin-Madison, Madison, USA; 2 Department of Microbiology and Infectiology, Universite de Sherbrooke, Sherbrooke, Canada

## Abstract

**Background:**

Cancer cells utilize a variety of mechanisms to evade immune detection and attack. Effective immune detection largely relies on the formation of an immune synapse which requires close contact between immune cells and their targets. Here, we show that MUC16, a heavily glycosylated 3-5 million Da mucin expressed on the surface of ovarian tumor cells, inhibits the formation of immune synapses between NK cells and ovarian tumor targets. Our results indicate that MUC16-mediated inhibition of immune synapse formation is an effective mechanism employed by ovarian tumors to evade immune recognition.

**Results:**

Expression of low levels of MUC16 strongly correlated with an increased number of conjugates and activating immune synapses between ovarian tumor cells and primary naïve NK cells. MUC16-knockdown ovarian tumor cells were more susceptible to lysis by primary NK cells than MUC16 expressing controls. This increased lysis was not due to differences in the expression levels of the ligands for the activating receptors DNAM-1 and NKG2D. The NK cell leukemia cell line (NKL), which does not express KIRs but are positive for DNAM-1 and NKG2D, also conjugated and lysed MUC16-knockdown cells more efficiently than MUC16 expressing controls. Tumor cells that survived the NKL challenge expressed higher levels of MUC16 indicating selective lysis of MUC16^low ^targets. The higher csMUC16 levels on the NKL resistant tumor cells correlated with more protection from lysis as compared to target cells that were never exposed to the effectors.

**Conclusion:**

MUC16, a carrier of the tumor marker CA125, has previously been shown to facilitate ovarian tumor metastasis and inhibits NK cell mediated lysis of tumor targets. Our data now demonstrates that MUC16 expressing ovarian cancer cells are protected from recognition by NK cells. The immune protection provided by MUC16 may lead to selective survival of ovarian cancer cells that are more efficient in metastasizing within the peritoneal cavity and also at overcoming anti-tumor innate immune responses.

## Introduction

Ovarian cancer is the deadliest of the gynecological malignancies. Eighty percent of the 14,000 cases of ovarian cancer that are diagnosed each year are of epithelial cell origin. Epithelial ovarian cancer is associated with the formation of a large amount of peritoneal fluid and is extremely metastatic. Immune regulation plays an important role in controlling ovarian tumor growth. Infiltration of T cells within the tumor is strongly associated with an increase in 5-year survival of ovarian cancer patients [1]. Primary cancer cells are known to express PVR and nectin, ligands for the activating NK cell receptor DNAM-1 [2,3]. Recognition of these ligands results in lysis of ovarian cancer cells by naïve NK cells present in the systemic circulation.

Ovarian tumors, however, have developed elaborate mechanisms to counter immune recognition and attack. Factors produced by the tumor can alter the expression of important activating molecules on immune cells present in the peritoneal cavity. In one study, a 10-14 kDa protein produced by the tumor cells was shown to downregulate the expression of the key signaling molecule CD3ζ [4]. Decreased expression of CD3ζ causes impairment of the immune response [4,5]. Macrophage migration inhibitory factor (MIF) produced by ovarian tumor cells decreases the transcription and expression of the activating receptor NKG2D on NK cells thereby inhibiting their ability to recognize and lyse ovarian tumor targets [6]. Other NKG2D ligands expressed by ovarian cancer cells include MICA, MICB, and Letal [7-9].

We have studied the effects of one particular factor produced in high quantities by the tumor cells, MUC16, and its effect on the cytolytic function of human NK cells [10,11]. MUC16 is a membrane spanning mucin with an average molecular weight between 3-5 million Da [12]. The high molecular weight of MUC16 is a result of the over 24,000 amino acids that constitute the protein backbone and also the extensive O-linked and N-linked glycosylation of this molecule [12-14]. Ovarian tumors present MUC16 as a Type I membrane glycoprotein on their cell surface. We refer to the cell surface bound mucin as csMUC16. Proteolytic cleavage at a site 50 amino acids upstream of the transmembrane region is hypothesized to result in shedding of the mucin from ovarian tumors [12,15,16]. The shed mucin, sMUC16, is present at considerable concentration (5-20 nM) in the peritoneal fluid and also leaks into the systemic circulation. csMUC16 and sMUC16 carry a repeating peptide epitope that has been previously characterized as the ovarian tumor marker CA125 [13,17].

sMUC16 is a potent inhibitor of the cytolytic ability of NK cells [11]. Incubation of NK cells from healthy donors with sMUC16 results in a 40-70% decrease in surface expression of CD16 [10,11]. Downregulation of CD16, a low affinity Fc receptor, impairs the ability of the peritoneal NK cells to mediate Antibody-Dependent Cell Mediated Cytotoxicity (ADCC) [5,18-22]. Thus sMUC16 directly inhibits the natural cytotoxicity mechanism of NK cells and may also indirectly attenuate ADCC in NK cells of ovarian cancer patients.

To date, csMUC16 has not been studied for its potential role in protecting ovarian tumor cells from immune attack. csMUC16, similar to sMUC16, may directly interact with NK cells and inhibit their ability to lyse tumor targets. Alternatively, csMUC16 may also protect ovarian tumor cells from NK attack by a different mechanism. Mucins are known to possess both adhesive and anti-adhesive properties [23]. csMUC16 acts as an anti-adhesive molecule in the endometrium and must be absent for successful trophoblast adherence to the endometrial wall [24]. MUC16 has also been shown to have a barrier function against pathogen adherence on corneal epithelial cell layers [25-27]. RNA interference of MUC16 expression in human corneal-limbal epithelial cells resulted in increased *Staphylococcus aureus *adhesion on the corneal epithelial cell layer [25].

Because NK cells form immune synapses with their target cells [28-30], which involve very close cell-cell contact, the presence of an anti-adhesive molecule on the surface of ovarian tumor cells may have significant consequences for tumor cell interactions with NK cells. The formation of these synapses allows NK cells to effectively read the inhibitory or activating ligands on the surface of the target cells. The strength and level of expression of these ligands determines the subsequent action of the NK cell [31]. The inability of NK cells to form these synapses would restrict effector cell activation and subsequent lysis of tumor targets.

Here, we provide evidence that the expression of csMUC16 attenuates the interactions between ovarian cancer cells and NK cells. Our data indicates that NK cells are unable to form immune synapses with the ovarian tumor targets, regardless of the expression of activating or inhibitory ligands on the surface of the tumor cells. This results in another redundant molecular mechanism that epithelial ovarian tumor cells utilize to avoid immune attack.

## Results

### NK cells preferentially target csMUC16^low ^OVCAR-3 cells

**Figure 1:**
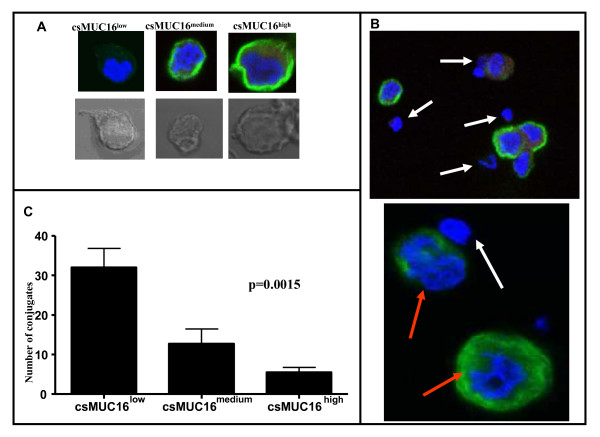
**Selective targeting of csMUC16^low ^OVCAR-3 cells by NK cells**. *A*, Upper panels show the classification of csMUC16^low, medium, and high ^OVCAR-3 cells. This classification was used to determine NK cell conjugation with different subsets of OVCAR-3 cells. The lower panel shows the corresponding bright field image of the cell in the upper panel. *B*, The upper panel is a 60× view of NK cells (white arrows) forming conjugates with csMUC16^low ^cells. NK cells surrounding csMUC16^high ^cells are not touching the tumor cell. The lower panel is a digital zoom image demonstrating selective conjugation of NK cells (white arrow) with csMUC16^low ^and not with csMUC16^high ^OVCAR-3 cells (red arrows). *C*, Conjugates of NK cells with low, medium and high csMUC16 expressing cells were quantified by averaging the number of conjugates observed per 50 fields. Data shown is mean of four independent experiments performed with NK cells from separate donors. A one-way ANOVA was performed and the resulting p value = .0015.

OVCAR-3 is an established ovarian tumor cell line that expresses csMUC16 and releases sMUC16 into the culture media. This cell line was also used for the cloning of MUC16 [17]. This cell line is therefore a good model to study the function of sMUC16 and csMUC16 [32]. NK cells derived from peripheral blood of healthy donors are unable to efficiently lyse OVCAR-3 cells [33,34]. To determine the role of csMUC16 in immune protection we conducted confocal microscopy experiments to analyze the direct interactions between OVCAR-3 cells and NK cells. The OVCAR-3 cell population exhibits a wide range of csMUC16 expression as determined by flow cytometry [35]. We developed an arbitrary scale to categorize OVCAR-3 as csMUC16^low^, csMUC16^medium^, and csMUC16^high ^based on fluorescent intensity as visualized by microscopy (Figure 1A).

OVCAR-3 cells were incubated with NK cells derived from peripheral blood of healthy donors. Invariably, we observed that NK cells selectively conjugated with csMUC16^low ^OVCAR-3 cells as compared to csMUC16^medium ^and csMUC16^high ^cells (Figure 1A, B). Quantification of NK cell-OVCAR-3 conjugates clearly showed that the effector cells formed 6-7-fold more conjugates with csMUC16^low ^OVCAR-3 cells compared to csMUC16^high ^OVCAR-3 (Figure 1C). Intermediate numbers of conjugates were observed between NK cells and OVCAR-3 cells expressing medium levels of csMUC16.

### csMU16^low ^cells express comparable levels of DNAM-1 and NKG2D ligands

One potential reason for differential binding between NK cells and csMUC16 low or high expressing OVCAR-3 cells could be that the csMUC16^low ^cells were expressing higher levels of ligands of the activating receptors DNAM-1 and NKG2D that have been shown to play an important role in lysing primary ovarian cancer cells [2,8,9,36]. To test this hypothesis, expression of DNAM-1 and NKG2D ligands on the OVCAR-3 cells was examined in relation to csMUC16 levels.

**Figure 2:**
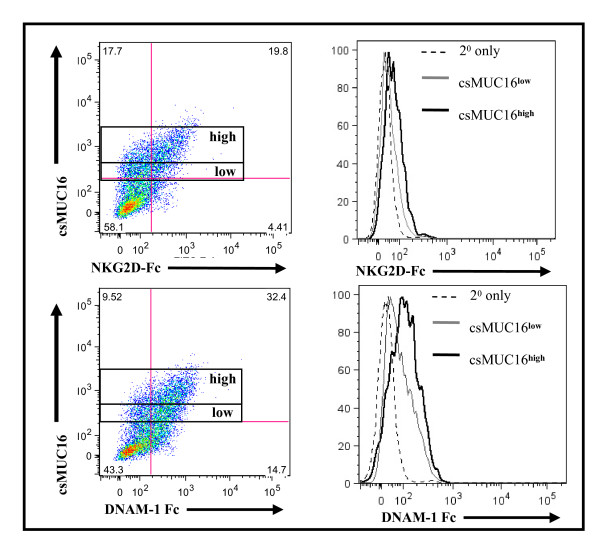
**DNAM-1 and NKG2D ligand expression is comparable on csMUC16^high ^expressing OVCAR-3 cells**. OVCAR-3 cells were stained for csMUC16 expression, and then NKG2D-Fc or DNAM-1-Fc and an appropriate secondary antibody were added. Cells were initially gated on live single events of tumor cells that were csMUC16^low ^or csMUC16^high^. The binding of NKG2D-Fc or DNAM-1 to gated csMUC16^low ^(gray line) and csMUC16^high ^(black line) is shown in histogram form. Data is representative of three independent experiments.

DNAM-1-Fc or NKG2D-Fc chimeras were added to OVCAR-3 cells and the cells were gated for high or low csMUC16 expression (Figure 2). OVCAR-3 cells expressing low or high csMUC16 expressed comparable levels of DNAM-1 and NKG2D ligands (Figure 2). These data indicate that differences in expression of DNAM-1 or NKG2D ligands was not a factor in the ability of NK cells to conjugate to OVCAR-3 cells.

### MUC16 knock-down OVCAR-3 cells

**Figure 3:**
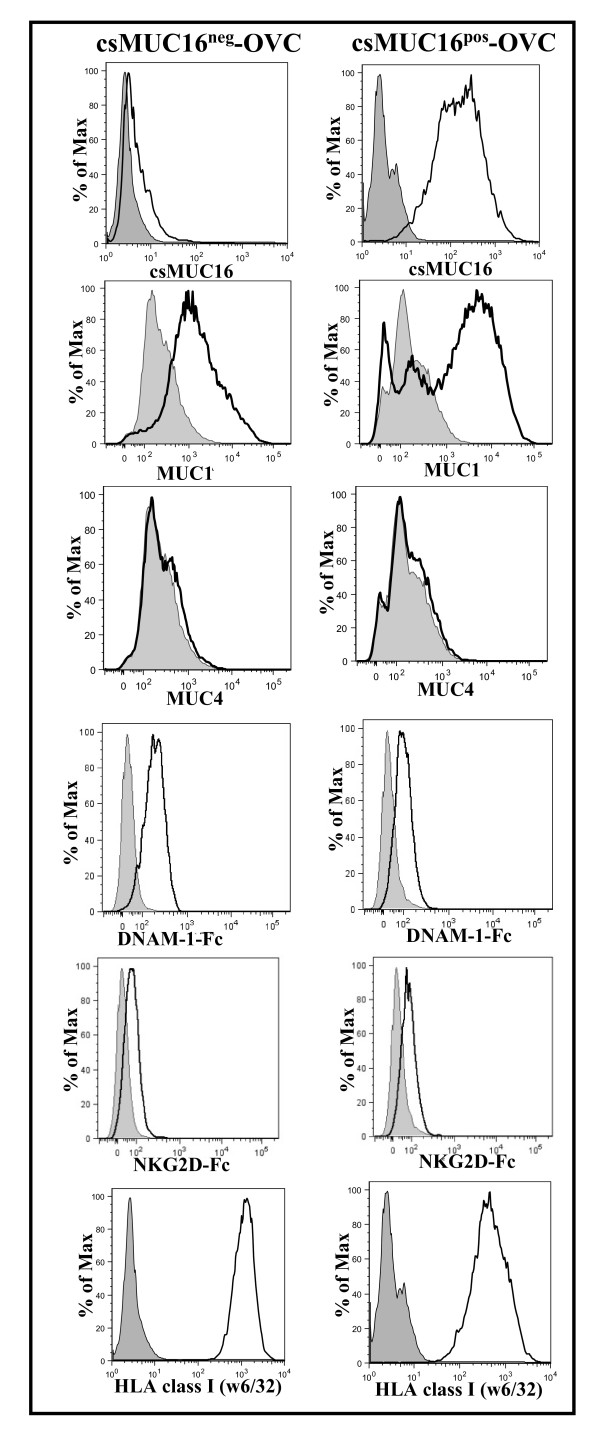
**Expression of mucins and NK cell activating and inhibitory molecules on csMUC16^neg^-OVC and csMUC16^pos^-OVC**. MUC1 expression on both sublines is similar and MUC4 expression is absent. Expression of DNAM-1 and NKG2D ligands on the sublines was determined by staining with Fc chimeras (15 μg/mL) of these two receptors followed by labeling with fluorophore conjugated anti-Fc secondary antibody. HLA class I expression on the sublines was determined by labeling with FITC conjugated w6/32. Histograms of live single events are shown in all plots. Data is representative of three independent experiments. Shaded peaks show control cells incubated with only the fluorescently-tagged secondary antibodies. Un-shaded histograms are for cells incubated with the primary antibodies or Fc chimeras and the fluorescently labeled secondary antibodies.

The significantly reduced ability of NK cells to bind to csMUC16^high ^OVCAR-3 cells suggested a potential role for this mucin in protecting ovarian cancer cells from immune recognition. To investigate this immuno-protective role, we employed MUC16 knock-down cells that were successfully utilized in our recent studies [35,37]. The subline csMUC16^neg^-OVC expresses an endoplasmic reticulum localized scFv fragment of the anti-MUC16 antibody VK-8 that prevents expression of csMUC16 and sMUC16. A control subline designated csMUC16^pos^-OVC that expresses an scFv fragment of an irrelevant murine IgG has no effect on the expression of csMUC16 and sMUC16 [37]. Expression of csMUC16 on both sublines is shown in Figure 3. The scFv-MUC16 complex is rapidly degraded in the proteasomes and the mucin does not accumulate in the cytoplasm of the tumor cells (see additional file 1). The csMUC16^pos^-OVC and csMUC16^neg^-OVC were negative for MUC4 expression and did not exhibit any major difference in the levels of MUC1 (Figure 3). This is important to note as it has previously been shown that both MUC1 and MUC4 exert anti-adhesive effects on immune synapse formation [38,39].

### Expression of DNAM-1 and NKG2D ligands and HLA class I on csMUC16^pos^-OVC and csMUC16^neg^-OVC

NKG2D and DNAM-1 ligand expression and presentation of HLA class I antigens on tumor cells are important factors that determine activation or inhibition of NK cells. csMUC16^neg^-OVC and csMUC16^pos^-OVC express comparable levels of DNAM-1 and NKG2D ligands, as determined by monitoring the binding of DNAM-1-Fc and NKG2D-Fc chimeras to these two sublines (Figure 3). Similarly comparable levels of MHC class I antigens were expressed by csMUC16^neg^-OVC and csMUC16^pos^-OVC (Figure 3).

### csMUC16 protects csMUC16^pos^-OVC from NK cell lysis

**Figure 4:**
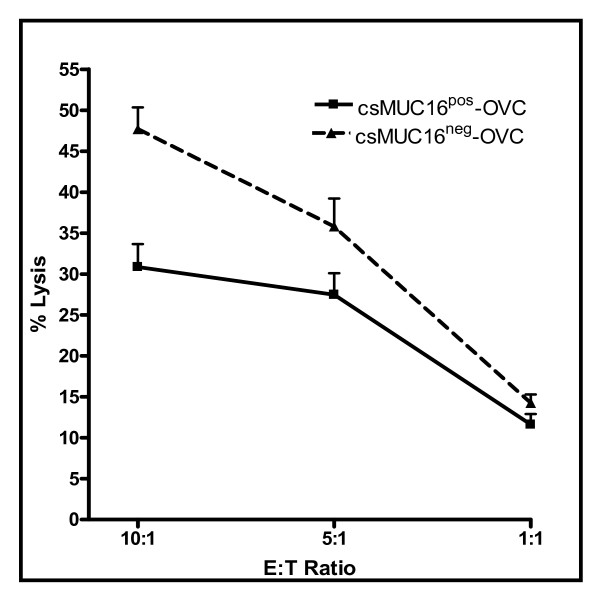
**csMUC16 protects ovarian cancer cells from lysis by NK cells**. NK cells from four healthy donors were isolated and ^51^Cr release lysis assays at 10:1, 5:1, and 1:1 effector:target ratios were conducted using csMUC16^neg^-OVC and csMUC16^pos^-OVC cells. Chromium release from the targets was determined after 4 h incubation under standard tissue culture conditions. Percent lysis was calculated by determining the spontaneous and maximum release of radioactivity from each cell line.

HLA class I antigens serve as strong inhibitory ligands of the Killer Immunoglobulin-like Receptors (KIR) of NK cells. Therefore, HLA class I expression protects tumor cells from NK cell attack. However, although comparable levels of HLA class I were expressed on the csMUC16^pos^-OVC and csMUC16^neg^-OVC the sublines exhibited differential susceptibility to NK cell mediated lysis. Higher protection of the csMUC16^pos^-OVC from NK cell mediated cytolysis as compared to the csMUC16^neg^-OVC was consistently observed (Figure 4). NK cells used in these assays were obtained from the peripheral blood of four healthy donors. Cytotoxicity assays at 10:1, 5:1 and 1:1 effector cell:target cell ratios showed that the csMUC16^neg^-OVC were lysed at a significantly higher level than the csMUC16^pos^-OVC (Figure 4).

### NKL cells lack KIR expression and are more effective at killing csMUC16^neg^-OVC

As shown in Figure 3, both the csMUC16^pos^-OVC and csMUC16^neg^-OVC expressed HLA class I antigens that could contribute to their protection from NK cell mediated lysis. Therefore, another effector cell model was required where the protection provided by HLA class I antigens would not be as pronounced as with the primary NK cells from healthy donors.

**Figure 5:**
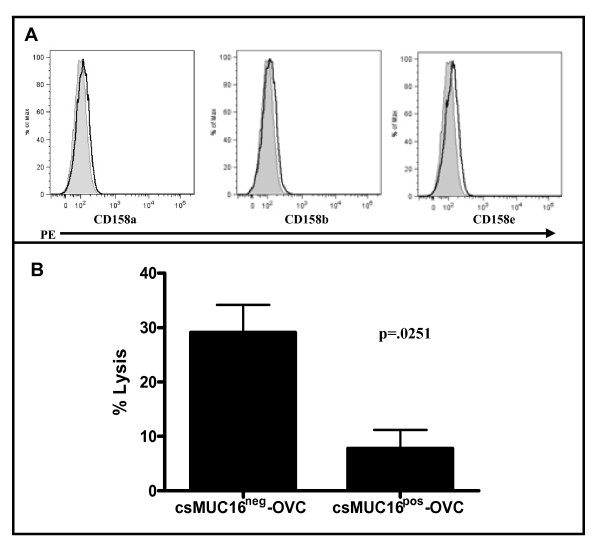
**NKLs lack KIRs and exhibit reduced ability to lyse csMUC16^pos^-OVC**. *A* Expression of KIR on NKL cells was determined by flow cytometry. *B*. Flow cytometry based cell lysis assays (24:1 effector:target ratio) were conducted to demonstrate that NKL cells lyse csMUC16^neg^-OVC cells to a greater extent than the csMUC16^pos^-OVC cells. Mean, standard deviation and p-value of three independent measurements is shown.

The NK cell leukemia cell line, NKL, does not express the prominent KIR (Killer Immunoglobulin-like Receptors) CD158a, CD158b, and CD158e (Figure 5A). These KIRs are inhibitory receptors that bind to HLA class I molecules and abrogate the cytotoxic responses of NK cells [40]. The lack of KIR on NKL cells therefore reduces the inhibitory effect arising out of a major pathway (HLA class I-KIR) displayed by the csMUC16^pos^-OVC and csMUC16^neg^-OVC sublines and allows better isolation of the immunoprotective properties of csMUC16.

To assess the ability of NKL cells to lyse either subline, flow cytometry based cytotoxicity assays were performed with these cells. There was a 2-3 fold increase in killing when NKLs were paired with csMUC16^neg^-OVC compared to csMUC16^pos^-OVC (Figure 5B).

### csMUC16^pos^-OVC form low numbers of conjugates with NK and NKL cells

**Figure 6:**
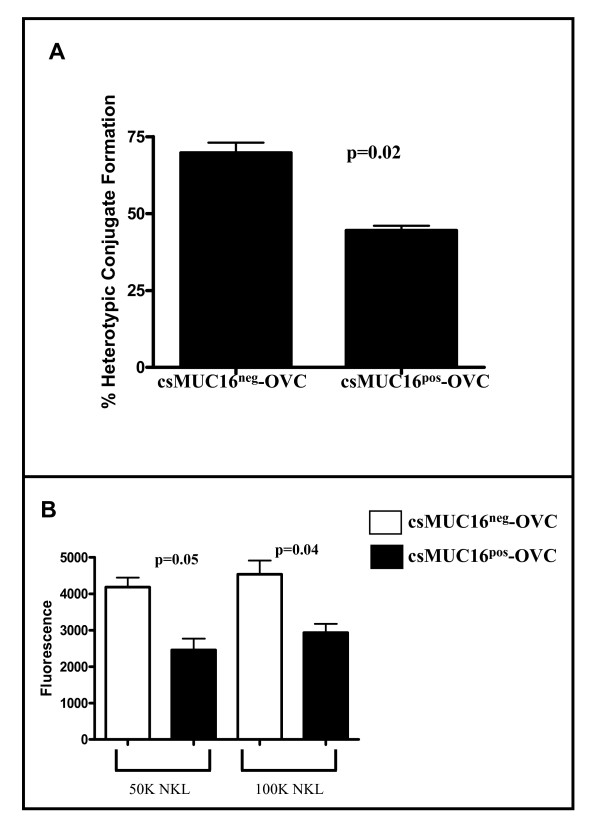
**csMUC16^neg^-OVC form more conjugates with NK and NKL cells than csMUC16^pos^-OVC**. *A*, NK cells from healthy donors were labeled with CellTracker Green and mixed with CellTracker Blue labeled csMUC16^neg^-OVC or csMUC16^pos^-OVC cells. Conjugation between the tumor cells and NK cells was determined by flow cytometry. Mean values of NK cell-tumor cell conjugation obtained from experiments conducted with three healthy donors are shown. *B*. csMUC16^neg^-OVC or csMUC16^pos^-OVC were plated in a 96-well plate and allowed to grow to 70-80% confluence. Calcein dyed NKLs were added to the plate. After incubation, non-adherent cells were removed by washing and the NKL cells bound to tumor targets were quantified using a fluorescence plate reader. Data for two experiments was combined for a total of 12 replicates.

Because NK cells were selectively binding to csMUC16^low ^OVCAR-3 cells (Figure 1), we determined if they also exhibited a higher ability to conjugate with csMUC16^neg^-OVC. We first quantified doublet formation between fluorescently tagged effector and target cells by flow cytometry. Approximately 1.5-fold more doublets were observed between NK cells (isolated from three different healthy donors) and csMUC16^neg^-OVC as compared to csMUC16^pos^-OVC (Figure 6A).

Similar to our observations with NK cells, we were able to demonstrate that csMUC16^neg^-OVC formed increased numbers of conjugates in plate-based adhesion assays with NKL cells compared to csMUC16^pos^-OVC (Figure 6B). Higher adhesion correlated with 2-3-fold higher killing of the csMUC16^neg^-OVC by the NKL cells (Figure 5B).

### csMUC16^neg^-OVC cells formed higher numbers of activating immune synapses with NK cells

**Figure 7:**
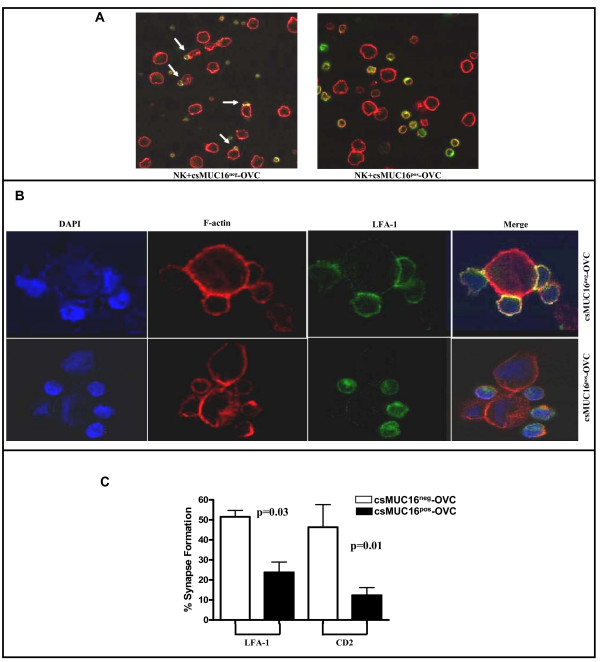
**csMUC16 inhibits NK activating synapse formation**. *A*. NK cells from healthy donors were mixed with csMUC16^neg^-OVC and csMUC16^pos^-OVC cells and confocal microscopy was conducted using 10× magnification. White arrows indicate NK cells bound to tumor cells and some forming activating immune synapses. *B*. Immune synapses between the NK cells and csMUC16^neg^-OVC at 40× magnification are shown. NK cells are present in the vicinity of csMUC16^pos^-OVC cells but form significantly less conjugates and immune synapses. *C*. Immune synapses between NK cells from three healthy donors and the csMUC16^neg^-OVC or the csMUC16^pos^-OVC cells were quantified by counting conjugates that showed polarization of LFA-1 and CD2 divided by the total number of conjugates (defined as two cells in contact).

Conjugation between NK cells and targets results in formation of immune synapses. NK cells form activating immune synapses in order to lyse target cells. We therefore determined if the increased conjugation between csMUC16^neg^-OVC and NK cells resulted in the formation of activating immune synapses. Activating immune synapses between NK cells and csMUC16^pos^-OVC and csMUC16^neg^-OVC were quantified by determining the polarization of LFA-1 or CD2 and F-actin at the interface between the effector and target cells. csMUC16^neg^-OVC cells formed twice as many activating immune synapses with NK cells compared to csMUC16^pos^-OVC (Figure 7). In some cases, multiple NK cells were also found to form simultaneous activating immune synapses with the csMUC16^neg^-OVC cells (Figure 7B). Although NK cells were found in the vicinity of csMUC16^pos^-OVC, activating immune synapses were not formed (Figure 7B).

### csMUC16 protects adherent tumor cells from NKL-mediated lysis

**Figure 8:**
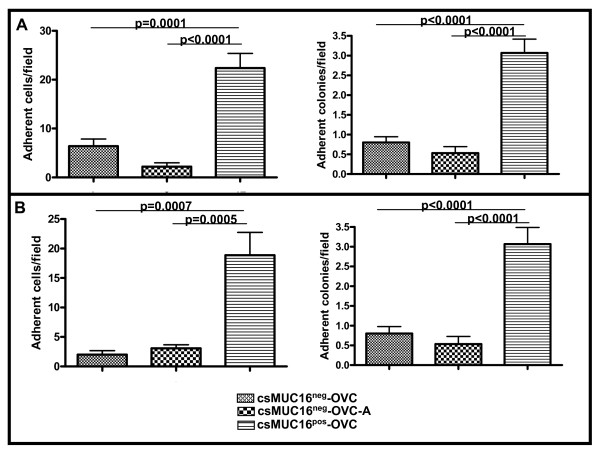
**csMUC16 shields ovarian cancer cells from NKL mediated lysis**. *A*. NKL cells were added to confluent cultures of csMUC16^neg^-OVC or csMUC16^pos^-OVC cells at a 1:1 effector:target ratio. After incubation for *A*, 24 h and *B*, 48 h the number of adherent colonies and adherent cells surviving the NKL challenge were counted by microscopic examination of five random fields. This experiment was repeated in triplicate and mean values (n = 15 fields) and standard deviation are shown.

The ability of NKL cells to selectively lyse csMUC16^neg^-OVC and csMUC16^pos^-OVC was further evaluated in co-culture experiments. An additional MUC16-knockdown clone (csMUC16^neg^-OVC-A) was also used in these experiments. This protocol for the generation of this additional cell line has been reported [37]. The three sublines were plated at equal densities in separate wells. After cells were 70% confluent, NKL cells were introduced in the cultures and plates were incubated for 24 h (Figure 8A) or 48 h (Figure 8B). At the end of the incubation, NKL cells and lysed non-adherent ovarian tumor cells were removed and the remaining adherent colonies and individual cells were manually counted. Very few adherent colonies and cells per observed field remained in the csMUC16^neg^-OVC/NKL and csMUC16^neg^-OVC-A/NKL co-cultures (Figure 8). However, 3-4-fold more colonies and cells/field were detected in the csMUC16^pos^-OVC/NKL co-cultures (Figure 8).

### NKL-resistant csMUC16^pos^-OVC express higher levels of csMUC16

**Figure 9:**
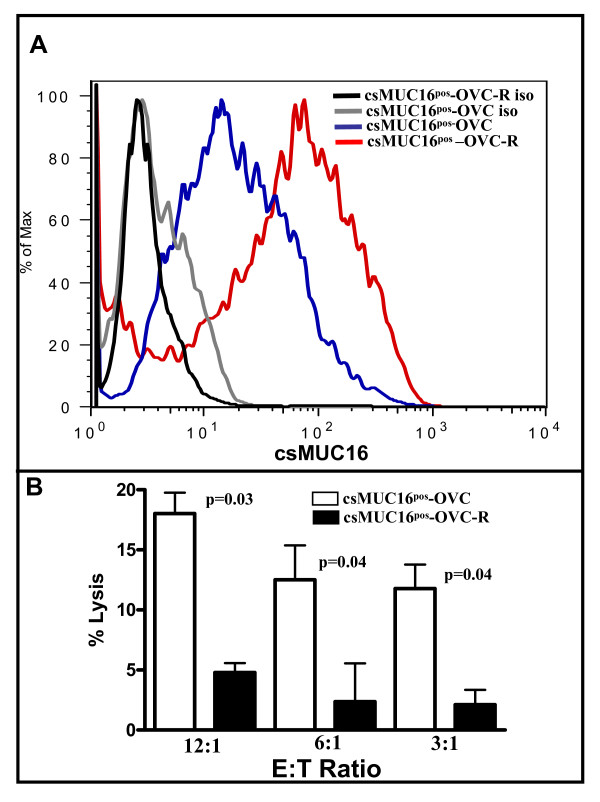
**Tumor cells surviving NKL challenge express higher levels of csMUC16**. *A*. csMUC16^pos^-OVC cells not treated with NKL or those surviving NKL treatment (csMUC16^pos^-OVC-R) were analyzed for csMUC16 expression by flow cytometry. Data is representative of three independent experiments. *B*, csMUC16^pos^-OVC and csMUC16^pos^-OVC-R were labeled with ^51^Cr. Cytotoxity assays were performed by using NKL cells as effectors. After 4 h co-incubation, released radioactivity was measured. Each bar is mean of four independent assays.

Adherent csMUC16^pos^-OVC cells that were resistant to two sequential NKL challenges (Figure 8) were designated as csMUC16^pos^-OVC-R. The surviving csMUC16^pos^-OVC-R cells were further cultured to 100% confluence and csMUC16 expression on these cells was determined by flow cytometry. Compared to csMUC16^pos^-OVC that were cultured at identical confluence and similar passage number, but had not been exposed to NKL cells, the csMUC16^pos^-OVC-R expressed 2-fold higher levels of csMUC16 (Figure 9A).

Based on all of the data obtained in this study we predicted that the csMUC16^pos^-OVC-R would be more resistant to killing by effector cells due to the even higher expression of csMUC16. Indeed, when NKL cells were used as effector cells, the csMUC16^pos^-OVC-R cells showed a 3-4-fold decreased susceptibility to lysis as compared to the csMUC16^pos^-OVC that had not been previously subjected to NKL treatment (Figure 9B). These data are consistent with results obtained from the cytotoxicity experiments using csMUC16^pos^-OVC and csMUC16^neg^-OVC and also support our initial observation that NK cells selectively form decreased numbers of conjugates with OVCAR-3 cells that express higher levels of csMUC16.

## Discussion

An NK cell lyses target cells based on the expression level and strength of activating and inhibitory signals. Expression of HLA class I antigens on ovarian tumor cells acts as a strong signal that abrogates the cytotoxic responses of NK cells by serving as ligands for the inhibitory KIR and LIR receptors. In addition, signal peptides of HLA class I antigens bind to the non-polymorphic HLA-E molecules [41]. The signal peptide-HLA-E complex is presented on the cell surface where it serves as a ligand for the inhibitory CD94/NKG2A receptor complex expressed on NK and other immune cells [42-44]. On the other hand, ovarian tumor cells also express ligands for the activating receptors NKG2D and DNAM-1 [2] that make them susceptible to NK cell mediated lysis.

Recognition of inhibitory or activating ligands requires NK cells to form close contacts with tumor cells. The formation of such cell-cell interactions is dependent upon the molecular landscape of the cell surface on both cell types. While adhesive molecules are required to formalize contacts between the cancer and effector cells, anti-adhesive molecules may hinder proper recognition of the tumors by NK cells. This study identifies csMUC16 as an inhibitor of NK cell/tumor cell contact thereby providing a mechanism for ovarian tumor cells to evade immune recognition.

NK cells isolated from peripheral blood of healthy donors preferentially form conjugates with OVCAR-3 cells that expressed lower levels of csMUC16 (Figure 1). We determined that the high degree of targeting of the csMUC16^low ^OVCAR-3 cells was not due to higher expression of NKG2D or DNAM-1 ligands. The role of csMUC16 in attenuating NK cell recognition of ovarian cancer cells was delineated by using the csMUC16 knockdown OVCAR-3 subline, csMUC16^neg^-OVC. The csMUC16^neg^-OVC and the matching control subline csMUC16^pos^-OVC are similar in their expression of NKG2D and DNAM-1 ligands. HLA class I levels were slightly elevated on csMUC16^neg^-OVC cells compared to csMUC16^pos^-OVC. These observations would suggest that csMUC16^neg^-OVC would be better protected from NK cell killing as compared to csMUC16^pos^-OVC. To the contrary, primary NK cells lyse the csMUC16^neg^-OVC to a significantly greater extent than the csMUC16^pos^-OVC. Similar effect was observed when the KIR^neg ^NKL cells were used as effectors. Because of the lack of KIR, the expression of HLA class I on the targets has a markedly reduced inhibitory effect on the cytolytic capacity of the NKL cells. Therefore, lower amount of lysis of the csMUC16^pos^-OVC observed in the NKL experiments is primarily due to the protection provided by csMUC16.

We have shown previously that sMUC16 binds specifically to a subset of NK cells in epithelial ovarian cancer patients. Our current data indicates that the receptor for sMUC16 is the inhibitory receptor Siglec-9 (Belisle et al manuscript in preparation). The binding of csMUC16 to Siglec-9 could be a mechanism by which csMUC16 is causing inhibition of NK cells. We have shown using cytotoxicity assays that NKL cells are also inhibited by MUC16 present on csMUC16^pos^-OVC cells. This is significant because NKLs are Siglec-9 negative and do not bind to sMUC16 (data not shown), indicating Siglec-9-csMUC16 binding may not play a role in the observed inhibition of cytotoxicity.

An alternate explanation for NK cell protection afforded by csMUC16 lies in the extraordinary biochemical properties of the molecule itself. Mucins are known to exhibit both adhesive and anti-adhesive properties. In cancer, the loss of apical expression of these large molecules and the subsequent distribution over the entire cell membrane has consequences for cell-cell interactions [45]. The large size of the molecules as well as the extensive O-glycosylation present on mucins contributes to their anti-adhesive properties. MUC16 contains a large number of core 2 type O-glycans [14]. The overexpression of core 2 type O-glycans on T cells has been implicated in disrupting cell-cell interactions between B and T cells, preventing cross-talk between the cells and impairing the humoral immune response [46].

MUC16 has a protein backbone of approximately 24,000 amino acids [12] and a high degree of glycosylation, indicating that it may extend a considerable distance out from the surface of the cell. Mucins such as MUC1, MUC4 and MUC16 have semi-flexible rod-like structures extending outwards from the cell surface [45,47]. It is estimated that a 28-mer peptide with O-glycan chains has a linear length of approximately 7 nm [47]. Based on these calculations, csMUC16, with an approximately 24,000 amino acid protein backbone, can be expected to extend up to 1-5 μm from the surface of ovarian tumor cells. The NK synaptic cleft requires apposition of the effector and the target cells over a distance of 10-55 nm [48]. Thus, ovarian cancer cells may specifically utilize the very large and heavily glycosylated csMUC16 molecule to prevent the conjugation and eventual binding to NK cells.

Other large molecules have been shown to interfere with cell-cell contact because of the bulky nature of their extracellular domains. CD43, which is abundantly expressed on T cells and is heavily glycosylated, is removed from the immune synapse before synapse formation between T cells and APCs [49]. Other researchers have shown that dendritic cells induce the polarization of MUC1 on T cells to sites opposing the T cell-dendritic cell interface [39]. Carraway and colleagues have conclusively demonstrated that MUC4, another large molecular weight mucin, shields cancer cells from lysis by the lymphokine activated killer cells due to its anti-adhesive properties [38]. As the expression of both MUC1 and MUC4 were similar on csMUC16^pos^-OVC and csMUC16^neg^-OVC, these mucins did not contribute to the differences in conjugation and lysis that were seen between these two sublines.

The targeting of the csMUC16^low ^ovarian tumor cells by NK cells may result in the selective survival of csMUC16^high ^cancer cells. Such a selective increase in csMUC16^high ^ovarian cancer cells is supported by our observation that csMUC16^pos^-OVC that were resistant to NKL attack expressed a higher level of csMUC16 (Figure 9A). Higher expression of csMUC16 on the surviving cancer cells may not only allow them to withstand NK cell killing, as demonstrated in Figure 9B, but also provide them an avenue to do so while expressing lower levels of HLA and thereby reducing their vulnerability to T cells.

Our data may represent an example of an immunoediting mechanism that allows progression and proliferation of ovarian tumors. NK cells may selectively eliminate csMUC16^low ^cells. The csMUC16^high ^cells surviving NK cell attack not only have the advantage of effectively evading immune attack but can also utilize csMUC16 to adhere to mesothelial cells at various sites within the peritoneal cavity [35]. The surviving csMUC16^high ^ovarian tumor cells may also be expected to shed higher amounts of sMUC16. We have now demonstrated that sMUC16 binds Siglec-9, an inhibitory receptor present on cytolytic NK cells in addition to subsets of B cells and a majority of monocytes (Belisle et al manuscript in preparation). Selective immunoediting of csMUC16^high ^ovarian tumors may therefore lead to tumors that have a higher potential to withstand immune attack and also to metastasize and proliferate in the peritoneal environment.

## Conclusion

We have conclusively shown that the expression of csMUC16 blocks the conjugation step of immune synapse formation of NK cells with ovarian cancer targets. This blockage prevents NK cell lysis of cancer cells. Immune protection afforded by csMUC16 should therefore be considered as another important mechanism that promotes tumor growth.

## Materials and methods

### Reagents and effector cell isolation

Unless otherwise stated, all chemicals were purchased from either Sigma or Fisher. NK cells were isolated from healthy donors using the RosetteSep (Stem Cell Technologies) NK Cell Isolation Kit protocol. Purity of the isolated NK cells was between 80 and 90% based on flow cytometry using anti-CD3, CD16, and CD56 antibodies. Antibodies against MUC1, CD2, CD3, CD16, CD56, CD158a, CD158b, CD158e, and HLA Class I (w6/32), were from BD Biosciences. Anti-LFA-1 and anti-MUC4 antibodies were from eBiosciences and Zymed Laboratories, respectively. OVCAR-3 cells were obtained from ATCC (Rockville, Maryland). OVCAR-3 csMUC16 knockdown sublines csMUC16^neg^-OVC and csMUC16^neg^-OVC-A and control subline csMUC16^pos^-OVC were obtained as described in our recent studies [37,35].

### Slide preparation and analysis for confocal microscopy

NK-target synapse experiments were conducted according to previously established protocols [30,50,51]. OVCAR-3, csMUC16^pos^-OVC, and csMUC16^neg^-OVC cells were cultured in the conditions prescribed for OVCAR-3 cells by ATCC. Target cells (7.5 × 10^4 ^cells) were mixed with NK cells at a 1:1 ratio in a total volume of 400 μl. The cells were centrifuged for 5 minutes at 100 × g and I incubated at 37°C at 5% CO_2 _for 25 minutes. The media was aspirated and cells were resuspended in PBS without calcium and magnesium and placed on poly-L-lysine coated coverslips. All subsequent procedures were performed at room temperature. Cells were fixed with 3% paraformaldehyde (Polysciences, Inc.) for 15 min, washed twice with 150 mM glycine, permeabilized with 0.1% Triton-X for 4 min, washed with PBS, and blocked overnight with PBS containing 5% goat serum.

Cells were stained with murine antibodies against LFA-1, CD2, or MUC16 (VK8 antibody kindly provided by Dr. Beatrice Yin) in 5% goat serum for 30 min. Coverslips were washed with 1 mL PBS, stained with a cocktail of goat anti-mouse FITC (Jackson ImmunoResearch), and rhodamine conjugated phalloidin (Invitrogen) for 30 min. Cells were washed with PBS, dipped in deionoized water, and mounted with DAPI containing mounting media (Invitrogen). After drying overnight, cells were visualized using the Bio-Rad Radiance 2100 MP Rainbow confocal microscope. Fifty conjugates between NK and tumor targets were counted on each coverslip. Conjugates were defined as an effector (NK cell) and target (cancer cell line) in contact with one another. Each conjugate was scored for polarization of the activating synapse markers LFA-1 and F-actin or CD2 and F-actin. Polarization was determined using two criteria, both of which had to be met to be defined as a synapse. These criteria were as follows: if greater than 70% of the fluorescence (of CD2 and F-actin or LFA-1 and F-actin) was noted to be at the interface between target and effector cell, and if there was a noticeable "flattening" of the membranes at the contact interface, than the synapse was deemed an activating synapse. Percent synapse formation was determined as: (number of conjugates showing polarization of activating synapse markers/total conjugates ×100).

### Flow cytometry antibody staining

For flow cytometric analysis, cells were washed two times in PBS containing 1% BSA by centrifugation at 300 ×g for 10 minutes at 4°C. Cells were labeled with primary and secondary antibodies for 30 min on ice, and washed with PBS containing 1% BSA in between antibody staining and prior to flow cytometry. In the DNAM-1-Fc and NKG2D-Fc binding experiments, OVCAR-3, csMUC16^pos^-OVC or csMUC16^neg^-OVC were blocked with Goat IgG (BD Biosciences) for 15 min., washed, and 15 μg/mL of either DNAM-Fc or NKG2D-Fc (R&D Systems) was added for 30 min on ice. Goat anti-human-Fc FITC (Jackson ImmunoResearch) was added for 30 min and washed. To determine mucin and HLA class I expression cells were treated with the primary antibodies. Where appropriate, cells were stained with goat anti-mouse PE (1:100, Jackson ImmunoResearch). Immediately before data acquisition on an LSRII (Beckton Dickinson) flow cytometer, the viability indicator PI or DAPI were added to each sample. Automatic compensation was applied. FlowJo software (v. 4.6.1, TreeStar) was used for analysis of the raw flow cytometry data, and comparison and statistical analysis (student t-test) of the data was done using GraphPad Prism software (v. 4, GraphPad Software, Inc.).

### Chromium-based cytotoxicity assays

csMUC16^neg^-OVC, csMUC16^pos^-OVC, or csMUC16^pos^-OVC-R were labeled with ^51^Cr in suspension. The targets were mixed with NK cells isolated from 4 healthy donors in 96-well plates at different effector:target ratios. After 4 h incubation, ^51^Cr released from the targets was measured and percent lysis was determined as described previously [52]. Comparison and statistical analysis (student t-test) of the data was done using GraphPad Prism software (v. 4, GraphPad Software, Inc.).

### Cytotoxicity Assays of adherent csMUC16^neg^-OVC and csMUC16^pos^-OVC cells

CellTracker Green (Invitrogen) labeled NKL cells were added to wells of 6-well plates that contained confluent csMUC16^neg^-OVC or csMUC16^pos^-OVC cells. After 24 h incubation, the media and the adherent cells were harvested using trypsin-EDTA, transferred to a tube, stained with PI, and analyzed for percentage of live target cells by flow cytometry on a BD LSR-II instrument. Comparison and statistical analysis (student t-test) of the data was done using GraphPad Prism software (v. 4, GraphPad Software, Inc.).

Lysis of adherent csMUC16^neg^-OVC and csMUC16^pos^-OVC cells by NKL cells was also measured by counting the number of surviving adherent tumor targets. These experiments were set up similarly to the flow cytometry based cytotoxicity assays except that NKL cells were not stained. Following co-culture, the floating NKL cells and dead tumor cells were removed by gentle washing and the number of adherent (considered live) cells and colonies were counted on an inverted microscope. The cell counts from three independent experiments were pooled and the averages (n = 15) were plotted. csMUC16^pos^-OVC cells that survived NKL challenge were further cultured to confluence and re-challenged with NKL cells. Tumor cells surviving this second NKL treatment were harvested and analyzed by flow cytometry for amount of csMUC16 expression.

### Flow cytometry conjugate formation assay

Freshly isolated naïve NK cells were labeled with 0.5 μM CellTracker Red (Invitrogen) for 25 minutes at 37°C in 5% CO_2_. Concurrently, csMUC16^neg^-OVC and csMUC16^pos^-OVC cells were trypsinized, washed, and dyed with 1.25 pM CellTracker Green (Invitrogen) in the same conditions. After washing off excess dye with PBS, cells were resuspended in PBS containing 1% BSA solution. NK cells were placed in flow tubes with either csMUC16^neg^-OVC or csMUC16^pos^-OVC cells at a 1:1 ratio and centrifuged for 2 minutes at 100 ×g. Tubes were incubated for 25 minutes at room temperature, and vortexing was avoided. Immediately before data acquisition on an LSRII (Beckton Dickinson) flow cytometer, viability indicator DAPI (BD Biosciences) was added to each sample. Comparison and statistical analysis (student t-test) of the data was done using GraphPad Prism software (v. 4, GraphPad Software, Inc.).

### Plate adhesion assays

csMUC16^neg^-OVC and csMUC16^pos^-OVC were plated at 100,000 cells/well of a 96-well plate and allowed to grow for 48 hours to confluence. On the day of the assay, NKL (natural killer leukemia) cells were dyed with calcein AM (1 ug/mL, Invitrogen) in 1% PBS-BSA for 30 min at 37°C, washed, and resuspended in media containing FBS at a concentration of 50,000 or 100,000 NK cells/50 uL. Fifty μL of dyed NKLs were added to each well containing the previously plated csMUC16^neg^-OVC or csMUC16^pos^-OVC. The plate was centrifuged at 400 ×g for 5 minutes and placed in the incubator for 25 minutes. The plate was washed gently with 1% PBS-BSA three times to remove non-adherent cells and then read using the Victor V-3 plate reader (Perkin Elmer). Comparison and statistical analysis (student t-test) of the data was done using GraphPad Prism software (v. 4, GraphPad Software, Inc.).

## Competing interests

The authors declare that they have no competing interests.

## Authors' contributions

JAAG conducted the majority of the experiments and helped in designing experiments, data analysis, and preparing the manuscript. MF and JAB conducted some of the flow cytometry experiments. SH conducted the cell binding experiments. HH and SP assisted in maintaining the cell lines and isolation of blood samples. MM and CR developed the MUC16 knockdown cell lines. AK assisted in monitoring MUC16 expression by the knockdown cell lines. JPC assisted in designing the experiments and data interpretation. MSP is the corresponding author and helped in study design, data interpretation and preparation of the manuscript. All authors read and approved the final manuscript.

## Supplementary Material

Additional file 1 **MUC16 is not detected in the lysates of csMUC16^neg^-OVC**. 1. Lysates of OVCAR-3, csMUC16^pos^-OVC, and csMUC16^neg^-OVC cells were analyzed by western blotting. MUC16 was detected by using VK-8 (anti-CA125) as the primary antibody. Actin was used as loading control. Click here for file
