# Supplementary figures and images for: MUC16 provides immune protection by inhibiting synapse formation between NK and ovarian tumor cells
Source: Mol Cancer. 2010 Jan 20;9:11. doi: 10.1186/1476-4598-9-11 (PMC2818693; doi:10.1186/1476-4598-9-11)

## Slide 1
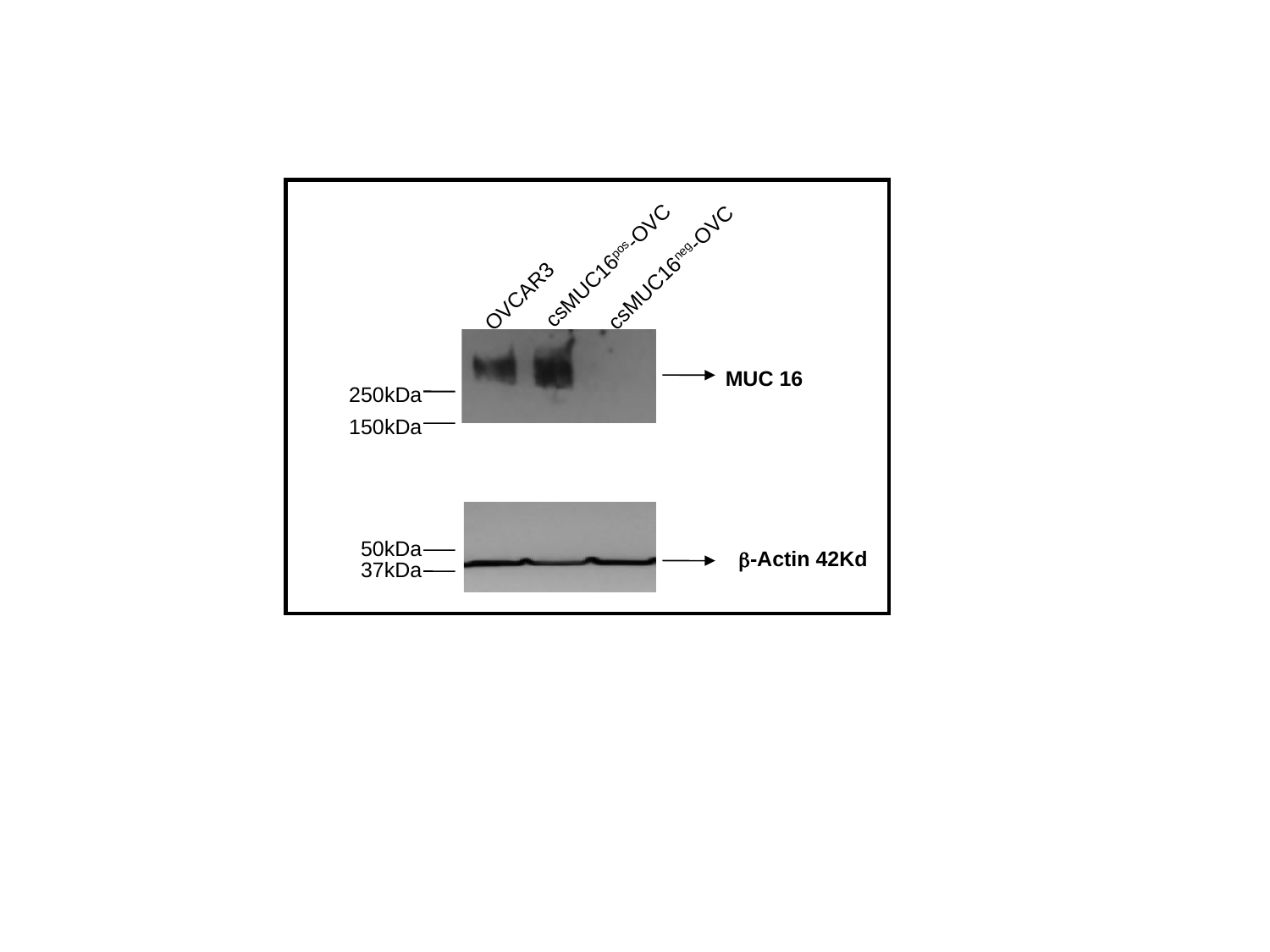

csMUC16pos-OVC
csMUC16neg-OVC
OVCAR3
MUC 16
250kDa
150kDa
 50kDa
β-Actin 42Kd
 37kDa

Supplement: Additional file 1 — MUC16 is not detected in the lysates of csMUC16neg-OVC. 1. Lysates of OVCAR-3, csMUC16pos-OVC, and csMUC16neg-OVC cells were analyzed by western blotting. MUC16 was detected by using VK-8 (anti-CA125) as the primary antibody. Actin was used as loading control. [file 1476-4598-9-11-S1.PPT]
